# Proteomics of Sarcopenia Prevalence and Future Risk: Shared and Distinct Pathways

**DOI:** 10.1093/geroni/igaf122.1221

**Published:** 2025-12-31

**Authors:** Hubert Leo, Aditya Surapaneni, Shoshana Ballew, Jordan Weiss, Morgan Grams, Marcus Goncalves, Josef Coresh

**Affiliations:** New York University, New York, New York, United States; New York University, New York, New York, United States; New York University, New York, New York, United States; NYU Grossman School of Medicine, New York, New York, United States; New York University, New York, New York, United States; New York University, New York, New York, United States; New York University, New York, New York, United States

## Abstract

Sarcopenia is a state of low muscle mass and function that is known to be related to systemic, low-grade, chronic inflammation. We screened over 5,000 proteomic measures to identify changes preceding sarcopenia incidence to inform development of therapies and risk stratification. We defined sarcopenia using the SDOC definition of weak grip strength and slow walking speed at the 2011–2013 (Visit 5, N = 6,538, mean (SD) age 76 (5)) and 2016-2017 (visit 6, N = 4,003) visits of the Atherosclerosis Risk in Communities (ARIC) study. We quantified aptamer measures of over 4,000 unique proteins from Visit 5 plasma using the SomaLogic platform. Sarcopenia was present in 665 participants (11.3%) at baseline and new incident cases developed in 391 (9.8%) by Visit 6. Among the 5,272 aptamers tested, 114 were associated with sarcopenia prevalence and 9 were associated with sarcopenia incidence after adjustment for age, sex, eGFRcr-cys, and multiple comparisons (Bonferroni p < 9.5E-6). The proteins associated with sarcopenia incidence (PXDN, TIMP4, RNASE1, GDF15, SVEP1, THBS2, SVEP1, ANGPT2, EGFR) were all associated with sarcopenia prevalence (eight at p < 4E-6; TIMP4 p = 1E-4). Pathway analysis on proteins associated with sarcopenia prevalence showed enrichment for Antigen Processing and Presentation, Apoptosis, Carbohydrate Digestion and Absorption, and Fanconi anemia pathways. Sarcopenia incidence prediction had an AUC of 0.713 for nine proteins, 0.709 for age and clinical factors, and 0.749 combined. This is the largest proteomic screen of incident sarcopenia and provides new insights into proteomic alterations that both precede and predict risk of sarcopenia.

